# Supplementary figures and images for: Patterns of change in nucleotide diversity over gene length
Source: bioRxiv. 2023 Jul 15:2023.07.13.548940. Preprint. [Version 1] doi: 10.1101/2023.07.13.548940 (PMC10369989; doi:10.1101/2023.07.13.548940)

Diversity

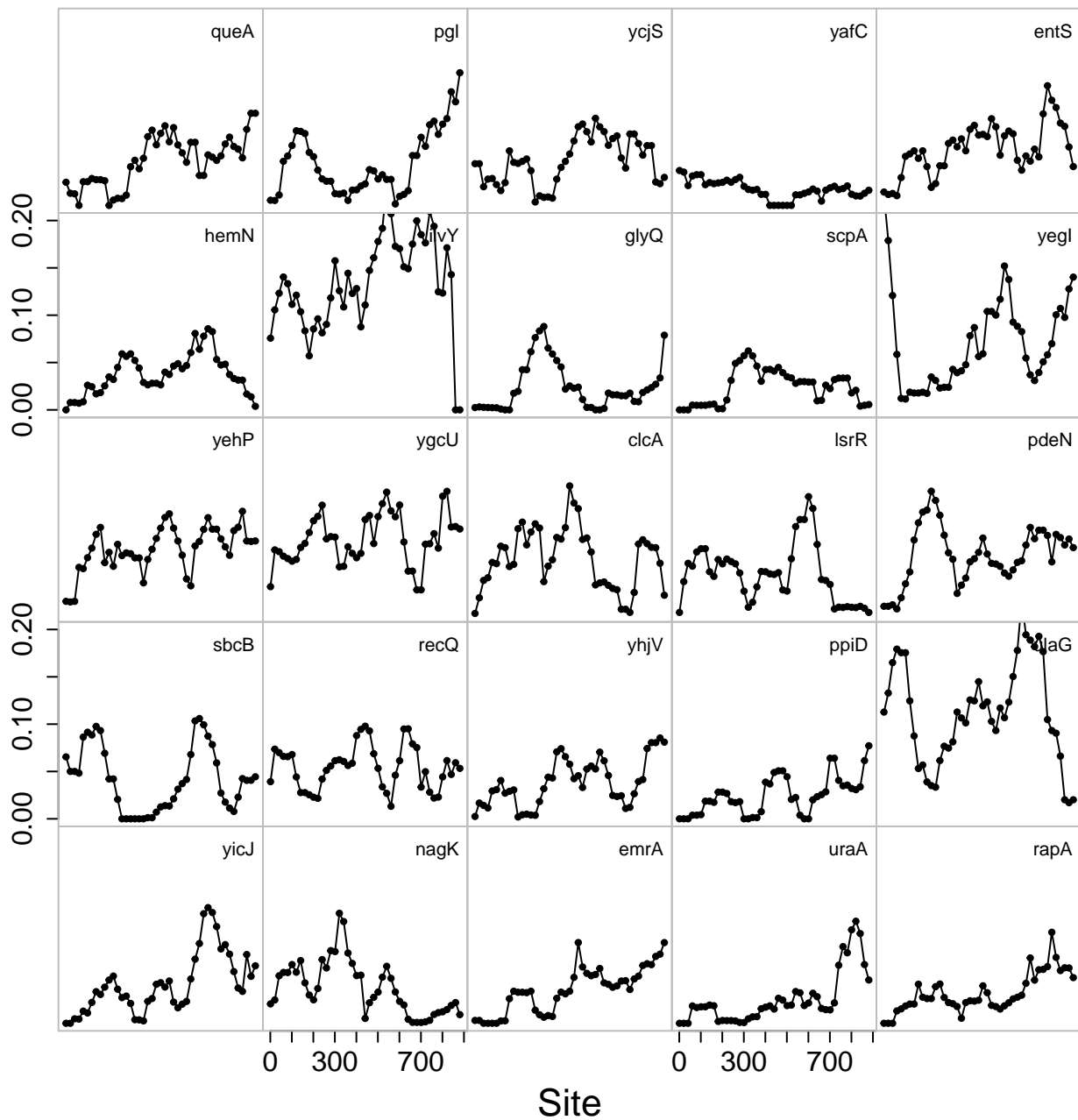

Supplement: Supplement 2 [file media-2.pdf]

Tree scale: 1

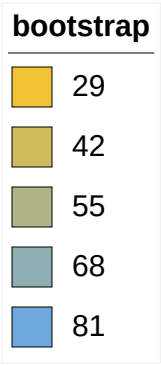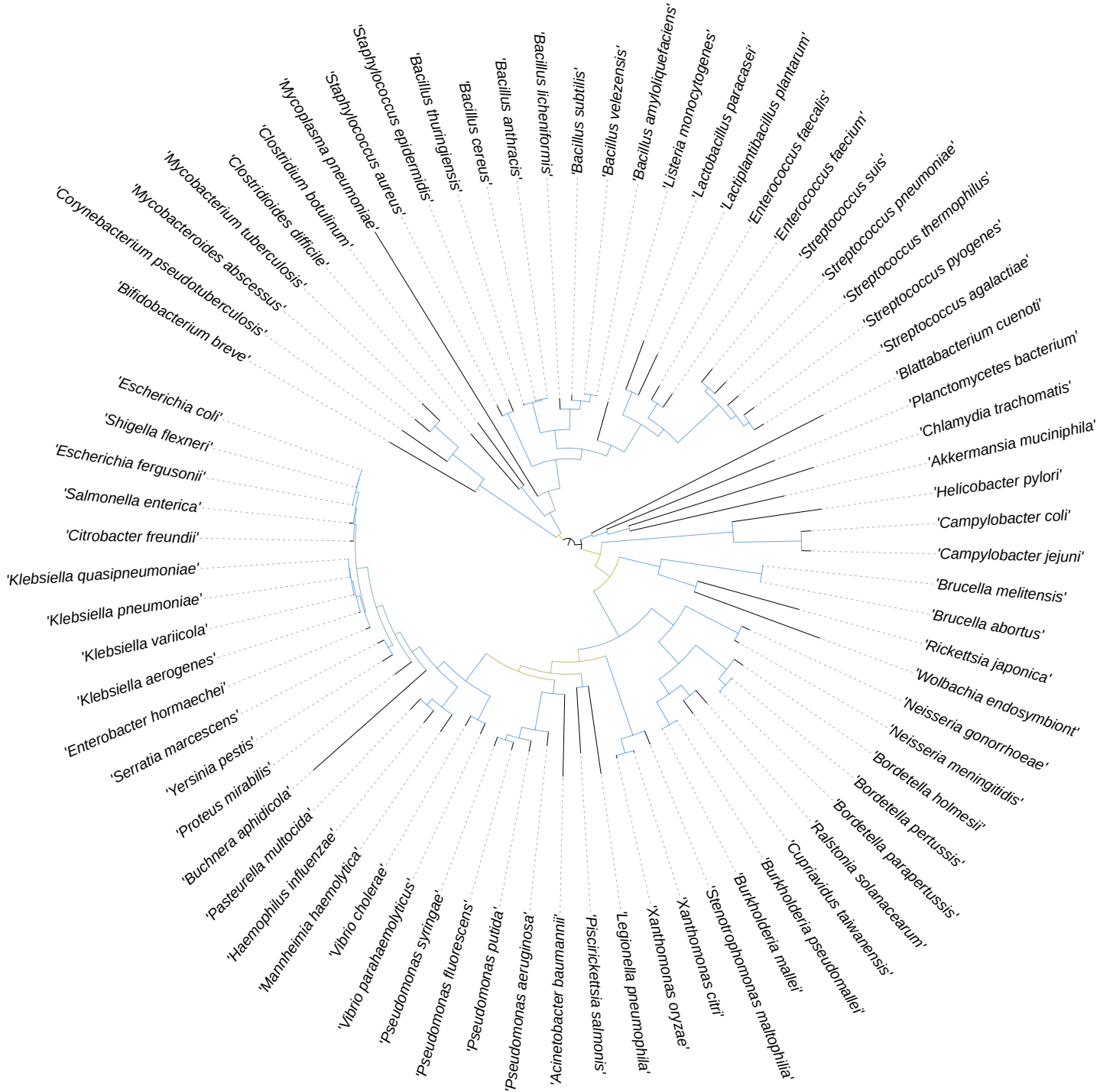

Supplement: Supplement 3 [file media-3.pdf]
